# Efficacy and influencing factors of the four‐step approach combining the situational simulation teaching method in the clinical practice of standardized training for residents

**DOI:** 10.1002/hsr2.757

**Published:** 2022-09-08

**Authors:** Tong Shan, Wang Kejun, Feng Ying, Huang Jia, Jiang Hongyan

**Affiliations:** ^1^ Center of Geriatrics Hainan Affiliated Hospital of Hainan Medical University (Hainan General Hospital) Haikou China; ^2^ Department of Academic Affairs Hainan Affiliated Hospital of Hainan Medical University (Hainan General Hospital) Haikou China; ^3^ Department of Otorhinolaryngology Hainan Affiliated Hospital of Hainan Medical University (Hainan General Hospital) Haikou China

**Keywords:** clinical practice, four‐step teaching method, situational simulation teaching method, standardized training for residents

## Abstract

**Background and Aims:**

Clinical skills practice is an essential component in standardized residency training. However, traditionally skill training methods are dogmatic and not all residents are exposed to such prescribed situations during their residency. The aims of this study were to evaluate the effectiveness and influence factors of a four‐step approach combining situational simulation teaching methods in clinical practice for residents.

**Methods:**

Enrolled all second‐year residents from the internal medicine base between May 2017 and May 2018 (*n* = 94), randomly divided into two groups. Forty‐eight residents were selected as experimental group, while the others 46 as the control group. Adopted traditional clinical practice method in the teaching and assessment of the control group, while used four‐step approach combining situational simulation teaching method in experimental group. We compared the theoretical and skill assessment scores in preclass and postclass. Conducted a satisfaction survey after class and analyzed the influencing factors of the teaching effect evaluation.

**Results:**

There were no significant differences in the theoretical and skill assessment scores between experimental group and control group at the beginning. After the class, both the average skill assessment and Direct Observation of Procedural Skills scores of the experimental group were higher than those of the control. Satisfaction survey findings identified that the experimental group expressed higher satisfaction. Logistic regression showed that educational background, “situational simulation mode helps to improve clinical skills training,” “helps to maintain attention during learning,” and “helps improve the ability to exercise analysis and solve problems” were the influencing factors of learners' satisfaction.

**Conclusion:**

The application of four‐step approach combining situational simulation teaching methods in the clinical practice of residents can significantly improve skills, thinking ability, decision‐making ability, and teaching satisfaction. Therefore, four‐step approach combining situational simulation teaching methods is worth promoting in teaching clinical skills for internal medicine residency training.

## 1 INTRODUCTION

Standardized residency training is necessary for all new medical graduates looking to work in a clinical capacity in China. Cultivated clinical skills training are vital components, to let the residents to competently carry out skill procedures in various clinical settings.^1^ Due to the current tight medical environment and improved teaching conditions, an increasing number of medical colleges have established clinical skill‐lab centers, providing simulated equipment for regular students to improve practical opportunities, although the utilization rate and effort are not optimistic. Skill‐lab training is nowadays part of the training programs of almost all medical faculties, which offer a protected, mistake‐forgiving training environment.^2^ But the traditional teaching of clinical skills is teacher‐centered and lectured‐focused, it is more dogmatic, and the content is fixed; this is not conducive to the cultivation of clinical comprehensive and practical ability training of students.

There are many new instructional approaches to convey clinical technical skills as described in the literature. Among these, Peyton's method is becoming increasingly prevalent in medical education, which is also known as four‐step method. It has also represented the standard instruction within training courses of the European Society of Cardiology in 2000. Originally, Peyton's method was to be used in a situation in which there is only one student per instructor. This approach is very limited in general medical education. In 2014, Nikendei et al.^3^ first attempted to modify Peyton's method for teaching clinical skills, where one instructor instructed several students. Since then, more and more medical colleges have tried to develop a one‐to‐many teaching method.^4^, ^5^, ^6^, ^7^

The training aim of the residents is not only “do the thing right” but also “do the right thing.” Additionally, not all residents are exposed to each prescribed situation during their residency. To solve this problem, most medical colleges use simulation teaching tools.^8^, ^9^ Simulation in education provided a changing active learning environment where students are safe to make mistakes. Simulation also simulates the ability to think critically and problem solve, which are the hallmarks of a resident.^10^

Therefore, some current problems should be solved: 1. How can clinical skills simulation equipment be used efficiently and reasonably? 2. How can the quality of the clinical training of residents be improved? 3. How do we cultivate the clinical thinking and decision‐making ability of clinical training such that the staff not only know “how to do,” but also know “why do this “ and “how to do it in different clinical environments”?

We hypothesized that the four‐step approach combining the situational simulation teaching method would be effective and well accepted by residents and would enhance skills retention over time. This study aims to incorporate a teaching method that combines four steps along with situational simulation and feedback on clinical skills instruction for residents. We evaluated the effectiveness of multiple disciplinary dimensions using pre, posttext, including theory and skill assessments and a satisfaction questionnaire survey. We also analyzed the factors affecting teaching effectiveness.

## 2 SUBJECTS AND METHODS

### 2.1 Subjects

We enrolled all second‐year residents from the internal medicine base of Hainan Affiliated Hospital of Hainan Medical University between May 2017 and May 2018 (*n* = 94). Randomly assigned residents (simple computerized random numbers) to experimental group (*n* = 48) or control group (*n* = 46).

Forty‐eight residents in the experimental group used four‐step approach combining situational simulation teaching method, while others 46 residents in the control group adopted a traditional clinical practice method. Residents in both groups had a medical bachelor's degree or above. In each experimental group and control group, there were respectively 26 and 30 males (average age of 25 ± 8 and 24 ± 10), 40 and 37 undergraduates, 8 and 9 master's degrees, and 30 and 28 social enrollment students. There were 18 targeted training students in each group. There was no statistically significant difference in the above basic conditions (*p* > 0.05). The learning time and progress of the experimental courses of the two groups were synchronous.

### 2.2 Teaching content

“Pleural cavity puncture operation of pleural effusion” was taught, with a duration of 120 min.

### 2.3 Research contents and methods

The experimental and control groups obtained theoretical knowledge of the face‐to‐face mode. The control group adopted the traditional skill teaching method, where the teacher was the center of instruction, and the lecture was the main teaching method. The student then operated on a simulated device. The teacher answered questions for students according to the actual problems encountered during the operation and while working. The experimental group used four steps combining situational simulation teaching methods and had feedback for each student. The teaching content and teachers of the experimental group and the control group were the same.

#### 2.3.1 Four‐step approach^11^

##### Preparation

The teacher told the students the content of the skills to be taught in advance. Then prepared the instruments and simulation equipment. Residents were divided into groups, with up to five peoples in each group. Assessing the skills of each student: if the skill is a new operation, the teacher guides and personally trains the student. If the operation is one that the students already know, the student is selected to teach as the lecturer, with the teacher as the supervisor. Clear purpose: the training purpose of this skill was made clear before teaching. Discuss the potential role of this skill: explained and discussed the equipment needed for this skill at the beginning. Set up a clinical site to conduct scenario exercises.

##### Process

Step 1 – “Trainer Demonstrate”: The trainer demonstrates the skill at a normal pace and without additional comments.

Step 2 – “Trainer Deconstruction”: The trainer demonstrates the respective skill while describing each procedural substep in detail.

Step 3 – “Trainee talks the trainer do”: The trainer performs the skill for a third time based on the substeps described by the trainee 1.

Step 4 – “Trainee does”: The trainee 1 performs the skill on his own.

##### Debriefing

Given teaching feedback after completing each operation:

A. Self‐evaluated: participants self‐evaluated themselves, indicated what was done well in the learning operation and what needed improvement.
B. Peer‐evaluated: Other learners evaluated the learners, pointed out what worked, and the points that needed improvement.
C. The teacher commented, pointed out the students' strengths and the areas that needed improvement, and once more pointed out the training purpose, the key points, and the plan to use when encountering some unexpected events.

#### 2.3.2 Circulation

Afterwards, the other trainees performed the abovementioned cycle one by one.

“Trainee do another trainee describe”: The trainee 1 performs Step 3 following instructions of trainee 2; The trainee 2 performs Step 4…

Cycle repeated in turn until the last trainee.

#### 2.3.3 Situational simulation

Wrote simulated cases according to the thoracentesis syllabus requirements in the preparation of the course and the knowledge points to be mastered. The case involved some clinical events: shortness of breath and pale complexion during the puncture, difficulty breathing after the puncture, or a rash during lidocaine injection. During the fourth step of the student's operation, the teacher asked questions and provided different scenarios. Students were asked to make judgements and give treatment measures.

### 2.4 Evaluation

Before the class, each resident was objectively assessed on theoretical knowledge and skills (the total score is 100 points) as the preclass grades. At the end of 20th month of residency, all residents must have a second stage exam (including theoretical and skill test). We chose the test results of Pleural cavity puncture operation in second stage exam as the postclass assessment grades.

The skill assessment consists of two parts: skill operation for 70% and Direct Observation of Procedural Skills (DOPS)^12^ for 30% (attached files).

We conducted a satisfaction survey on students after the course to learn the students' attitude and evaluation of the four‐step approach combined with situational simulation. A questionnaire survey was conducted on the influencing factors of satisfaction in the experimental group. The questionnaire was homemade according to literature and expert advice. Revised regarding the opinions given by multiple teachers and students. The final questionnaire mainly included the learner's basic information, whether they are satisfied with the teaching mode and the possible impact of satisfaction. The questionnaire of “students were satisfied with the teaching model” refers to the Likert scale, which is divided into five levels: “very satisfied, satisfied, uncertain, dissatisfied, very dissatisfied.” The questionnaire concerning the possible influencing factors of satisfaction was divided into “satisfaction and dissatisfaction.” Each question had three options: “agree, uncertain, and disagree.” The 13 issues that may affect satisfaction included gender, source of students (commissioning, social recruitment), speciality (internal medicine, infectious department, other), education (master, undergraduate), “the four‐step approach is helpful for learning skills,” “the situational mode teaching method is helpful for learning skills,” “helps maintain attention during learning,” “helps improve learning efficiency,” “helps improve learning enthusiasm and initiative,” “helps combine theory with practice,” “helps improve the ability to analyse and solve problems,” “improves clinical workability,” and “improves autonomous learning ability.”

### 2.5 Statistical analysis

The data and the questionnaire were entered into Epidata 12.0 using two‐person independent entry. Two independent sample *t*‐tests were used to compare the grades. The rank‐sum test (Jonckheere‐Terpstra) of the ordered data of two independent samples was used to compare teaching satisfaction. A single factor logistic regression was performed on satisfaction and possible influencing factors, and then a multivariate logistic regression was performed on the variables with statistical significance. The significance test level was bilateral *p* < 0.05. Using Cronbach's *α* coefficient as the reliability index for the questionnaire, Cronbach's *α* coefficient should reach above 0.7. Statistical analyses were performed using SPSS (version 22.0, IBM).

## 3 RESULTS

### 3.1 Basic information

Compared with the control group, the basic information of the experimental group was not significantly different (*p* > 0.05): sex ratio, age, education level, enrollment type, or theoretical and operating scores before training (80.35 ± 11.2 vs. 85.78 ± 18.8; 66.05 ± 7.4 vs. 62.32 ± 10.4).

### 3.2 Theories and operational assessment of clinical skills after learning

Compared with the control group, skill operation assessment results in the second stage exam of the experimental group were significantly improved (96.34 ± 10.1 vs. 84.26 ± 9.6, *p* < 0.05); The difference in theoretical results was not statistically significant (95.12 ± 7.8 vs. 94.78 ± 10.8, *p* > 0.05). The average DOPS scores in the experimental was high than control groups in second stage exam (25.39 ± 2.18 vs. 18.83 ± 1.98, *p* < 0.05). The differences in the grasp of indications, communication with patients, preparation before the operation, precautions during the operation, postoperative treatment, and overall performance were statistically significant (*p* < 0.05) (Table 1).

**Table 1:** Objective skill scores in second stage exam

Type	Control group (*n* = 46)	Experimental group (*n* = 48)	*p*
Theoretical score (total 100 points)	95.78 ± 10.8	94.12 ± 7.8	0.090
Skill score (total 100 points)	82.5 ± 3.2	94.26 ± 9.6	0.010
Operation score (70 points)	58.77 ± 11.1	65.12 ± 10.6	0.006
DOPS (30 points)	18.83 ± 1.98	25.39 ± 2.18	<0.001
Indications	4.78 ± 0.97	6.32 ± 0.64	<0.001
Communicate	5.98 ± 0. 32	6.25 ± 0.44	0.008
Preparation before operation	6.08 ± 0.42	4.78 ± 0.17	0.030
Cautions during operation	5.72 ± 0.37	6.69 ± 0.45	0.012
Postprocessing	5.65 ± 0.12	5.98 ± 0.35	0.020
Overall performance	4.89 ± 0.87	5.18 ± 0.86	0.010

Abbreviation: DOPS, Direct Observation of Procedural Skills.

### 3.3 Satisfaction analysis

Forty‐eight and 46 questionnaires were distributed to members of the experimental group and the traditional group, and 94 copies were collected and entered, with an effective recovery rate of 100%. Through reliability analysis, the satisfaction questionnaire had good internal reliability, with Cronbach's *α* = 0.712. Comparison of teaching satisfaction

Teaching satisfaction of the experimental teaching group was better than that of the control group, and the difference was statistically significant (*p* < 0.05) (Table 2).

**Table 2:** Comparison of teaching satisfaction *n* (%)

Group	Very satisfied	Satisfied	Basically satisfied	Dissatisfied
Control group	14 (30.4)	12 (26.1)	8 (17.4)	12 (26.1)
Experimental group	24 (50.0)	18 (37.5)	2 (4.1)	4 (8.3)
*Z*‐value		−2.846		
*p*‐Value		0.004		

#### 3.3.1 Analysis of satisfaction and influencing factors

In the experimental group, 91.6% (44/48) of the residents expressed satisfaction when the four‐step teaching method was combined with the situational simulation teaching method, while the remaining 8.4% expressed dissatisfaction.

Logistic regression showed that educational background (*p* = 0.043), major (*p* = 0.016), “the situational simulation mode helps improve clinical skills training” (*p* = 0.001), “helps maintain attention during learning” (*p* = 0.011), “helps improve the motivation and initiative of learning” (*p* = 0.004) and “helps improve the ability to exercise analysis and solve problems” (*p* = 0.001) were the influencing factors in learner satisfaction. The forward multivariate binary classification logistic stepwise regression results showed that four variables were selected into the regression model: educational background (*p* = 0.009), “situational simulation mode helps improve clinical skills training” (*p* = 0.004), “helps maintain attention during learning” (*p* = 0.041) and “helps improve the ability to exercise analysis and solve problems” (*p* = 0.010). As shown in Table 3, undergraduate students' satisfaction was lower than that of postgraduate students (odds ratio [OR]: 0.002; 95% confidence interval [CI]: 0.001–0.118). Students who disagree with the “situational model helps improve clinical thinking” were less satisfied (OR: 0.001; 95% CI: 0.001–0.122). Students who agreed with “helps maintain attention during learning” are more satisfied (OR: 0.048; 95% CI: 0.005–1.012). Finally, students who agreed with “helps improve the ability of exercising analysis and problem‐solving” indicated high satisfaction (OR: 0.014; 95% CI: 0.001–0.212) (Table 3).

**Table 3:** Logistic regression of factors influencing satisfaction

Type	Univariate logistic regression		Multivariate logistic stepwise regression		
	*p*	OR (95% CI)	*β*	*p*	OR (95% CI)
Educational					
Master		1.0	_	_	_
Undergraduate	0.013	0.031 (0.024–0.178)	−6.13	0.009	0.002 (0.001–0.118)
Profession					
Internal medicine		1.0			1.0
Infectious diseases	0.024	0.147 (0.017–0.254)	_	_	_
Others	0.036	0.098 (0.012–0.198)	_	_	_
Situational model helps improve clinical thinking					
Agree		1.0			1.0
Uncertain	0.013	0.018 (0.002–0.441)	−3.12	0.019	0.031 (0.003–0.512)
Disagree	0.001	0.019 (0.003–0.234)	−7.04	0.004	0.001 (<0.001–0.122)
Helps maintain attention during learning					
Agree		1.0			1.0
Uncertain	0.023	0.078 (0.001–0.487)	−1.67	0.047	0.064 (0.007–0.943)
Disagree	0.011	0.021 (0.017–0.389)	−2.14	0.041	0.048 (0.005–1.012)
Helps improve learning motivation and initiative					
Agree		1.0	_	_	_
Uncertain	0.023	0.077 (0.0241–0.462)	_	_	_
Disagree	0.004	0.018 (0.001–0.211)	_	_	_
Helps improve the ability of exercise analysis and problem‐solving					
Agree		1.0			1.0
Uncertain	0.022	0.026 (0.021–0.746)	−2.13	0.037	0.044 (0.011–0.912)
Disagree	0.001	0.013 (0.002–0.118)	−5.14	0.010	0.014 (0.001–0.212)

## 4 DISCUSSION

The purpose of standardized training is to cultivate comprehensive clinicians with excellent theoretical knowledge, proficient clinical ability, strong communication skills, and noble medical ethics.^13^, ^14^ Among them, mastering clinical skills is more important and difficult.^1^ Clinical skills are still mainly taught by teachers, assisted by simulated teaching aids and bedside guidance in China. But the clinical situation is mutable, and various complicated problems may arise in clinical work. Therefore, how to let students master clinical skills in a short time with some level of flexibility is a difficult teaching problem.

“What students should do is more important than what teachers do in the course of studying.” A four‐step approach is a method of clinical practice teaching based on this concept. In teaching work, students should be the main body, and the teacher's role is as a complete helper, mentor, promoter, and organizer. Many teachers explored the four‐step approach in the teaching of clinical skills.^5^, ^15^, ^16^ However, the four‐step approach also has deficiencies in clinical teaching applications. Some nonoperational content is easily overlooked during instruction, such as preparation before surgery and humanistic care for patients. Situational simulations can enhance students' interest and improve their clinical decision‐making and communications abilities.^9^, ^17^, ^18^ We designed different clinical scenarios during instruction, which improved the students' enjoyment while training the clinical cogitation and decision‐making ability. Combining the requirements of “standardized training content and standards for resident physicians,” we tried the four‐step approach combined with the situational simulation teaching method to teach chest puncture.

We used operational assessment results combined with DOPS scores to evaluate clinical skill levels. The DOPS is a formative evaluation tool with a teaching function that pays more attention to evaluating clinical operation ability.^5^ The evaluator can directly observe the student's skill operation process and then immediately give evaluation and feedback. Compared with the traditional teaching method, the research results showed that the four‐step teaching method combined with the scenario simulation teaching method could effectively improve academic clinical skills and achieve better satisfaction. The analysis reasons are as follows: (1) in the four‐step teaching method, the student can be a teacher, discovering and asking questions, and guiding the unskilled learner toward mastering the knowledge; (2) the students who have been familiar with the skills can discover their own skills and weaknesses, and accept those found by other students and teachers through clinical skill training and 360° comprehensive information feedback^19^; (3) students can better master the knowledge points and cultivate the ability to solve the problems when they are combined with different clinical scenarios; and (4) students easily increase their learning concentration and efficiency through sharing experiences and evaluations.

In the analysis of satisfaction, we found that academic education, scenario‐mode teaching methods, and experimental teaching methods help improve the ability to analyze and solve problems. The above three points mostly affect satisfaction. The reasons for this were as follows: (1) Master's students have a stronger self‐learning ability than undergraduate students and are prone to play an active role in teaching; (2) some students have an insufficient grasp of operational skills and knowledge and insufficient experience to deal with a clinical scenario simulation; and (3) some students cannot correctly judge situational scenarios because of a lack of clinical knowledge. Maintaining attention during the learning process is also an influencing factor of satisfaction. The students who do not follow the teacher's rhythm during the teaching process feel that there is “nothing to do.” Based on these issues, four‐step teaching combined with situational simulation is more suitable for students with certain clinical knowledge and skills. In future teaching processes, we should pay attention to mobilizing the enthusiasm of other students, such as conducting peer evaluations appropriately.

## 5 LIMITATIONS

There were several limitations in our study. The questionnaire was prepared by the authors and used for the first time in this study. The small size of the group of students practicing the study with only one skill being evaluated. The evaluation of the approach is limited to student feedback, which is a significant limitation of this paper.

## 6 CONCLUSIONS

In summary, the combination of the four‐step and scenario simulation teaching methods would improve operating skills, clinical decision‐making abilities and enhance teaching satisfaction. According to different students, the formulation of syllabuses, the preparation of preview profiles before teaching new skills, active communication and discussion between students and teachers, and preview theoretical training for learners can all improve learning effectiveness for students.

## AUTHOR CONTRIBUTIONS

**Shan Tong**: Conceptualization; data curation; formal analysis; investigation; methodology; project administration; software; writing—original draft; writing—review & editing. **Kejun Wang**: Data curation; formal analysis; investigation; project administration. **Ying Feng**: Data curation; software. **Jia Huang**: Project administration. **Hongyan Jiang**: Conceptualization; funding acquisition; supervision; validation; visualization; writing—review & editing.

## CONFLICT OF INTEREST

The authors declare no conflict of interest.

## ETHICS STATEMENT

This study was certified by the Ethics Committee of Hainan Affiliated Hospital of Hainan Medical University. The questionnaire and group interviews had detailed explanations about the investigation's background and purpose, and all of participants were willing to participate.

## TRANSPARENCY STATEMENT

The lead author [Jiang Hongyan] affirms that this manuscript is an honest, accurate, and transparent account of the study being reported; that no important aspects of the study have been omitted; and that any discrepancies from the study as planned (and, if relevant, registered) have been explained.

## Supporting information

Supporting information.

Supporting information.

## Data Availability

The data supporting the findings of this study are available from the corresponding author upon reasonable request. The corresponding author had full access to all of the data in this study and takes complete responsibility for the integrity of the data and the accuracy of the data analysis.
