# Supplementary figures and images for: Efficacy and influencing factors of the four‐step approach combining the situational simulation teaching method in the clinical practice of standardized training for residents
Source: Health Sci Rep. 2022 Sep 8;5(5):e757. doi: 10.1002/hsr2.757 (PMC9455944; doi:10.1002/hsr2.757)

## Instructional Design

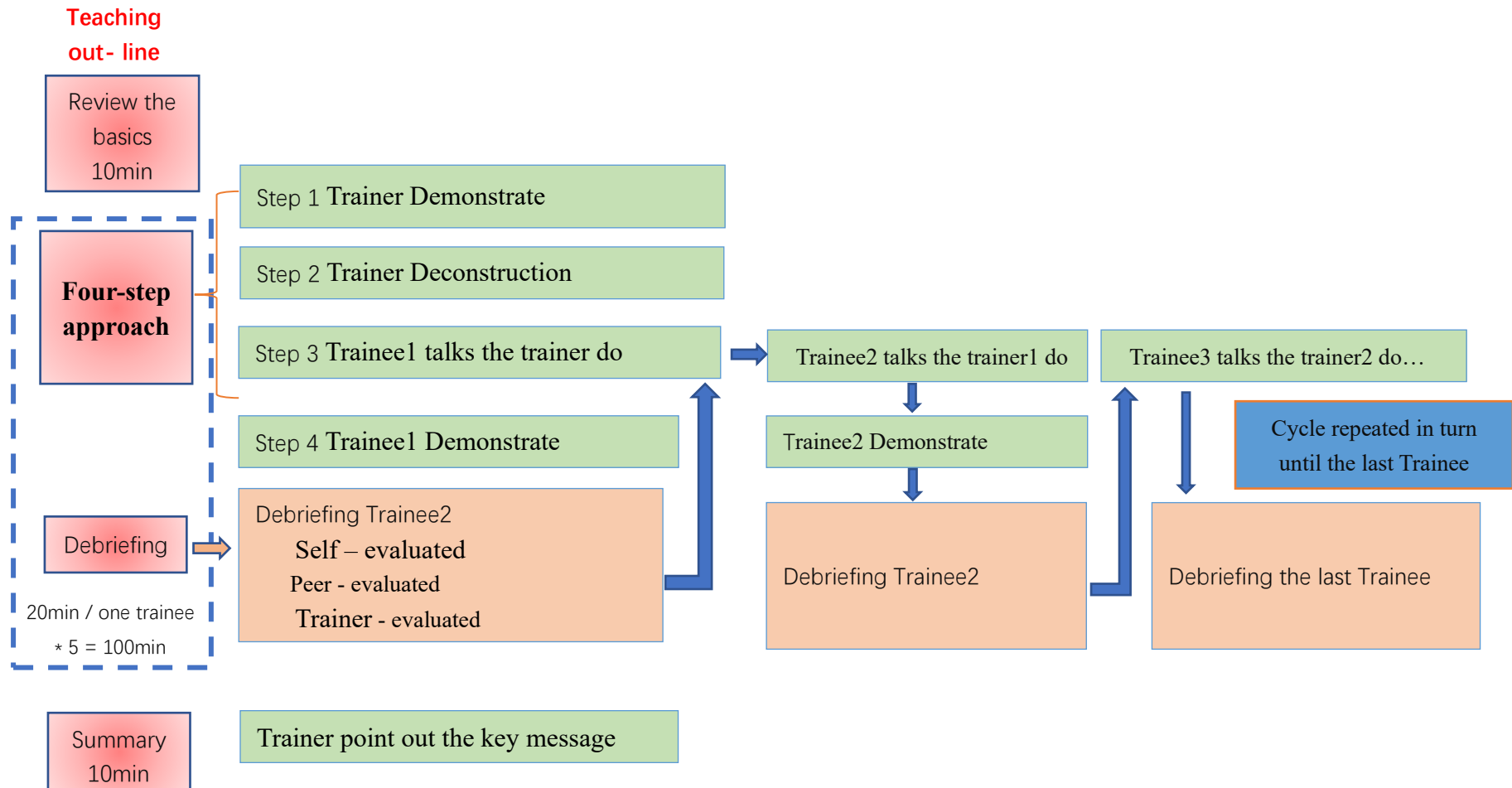

Supplement: Supplementary file 1 — Supporting information. [file HSR2-5-e757-s002.pdf]
