# Supplementary material for: Efficacy and influencing factors of the four‐step approach combining the situational simulation teaching method in the clinical practice of standardized training for residents
Source: Health Sci Rep. 2022 Sep 8;5(5):e757. doi: 10.1002/hsr2.757 (PMC9455944; doi:10.1002/hsr2.757)
Supplement: Supplementary file 2 — Supporting information. [file HSR2-5-e757-s001.pdf]

FROM 1 胸膜腔穿刺操作评分表 Evaluation form

name: score:

object		scoring criteria	Total score	result	Reason for deduction
Pre-	For patient	Measure vital signs, explain relevant content, sign informed consent	5		
	For material	Thoracentesis bag, medicines (Aner iodine or iodine-alcohol, 2% lidocaine, (Valium, 0.1% epinephrine)), others such as test tubes, sterile gloves	5		
	For operator	Wash hands, wear hat and mask, understand the patient's condition, the purpose of the puncture, and the chest X-ray et al.	5		
procedure	puncture point	1. Body position: a. Sitting position: facing the back of the chair, with the forearm on the back of the chair, and the forehead lying on the forearm; b. Semi-recumbent position: lying on the back on a high slope, slightly turning the affected side to the healthy side, and raising the pillow on the forearm on the affected side (4)<br>2. Puncture point: usually the 5th intercostal space of the anterior axillary line, the 6th to 7th intercostal space of the midaxillary line, the 7th to 8th intercostal space of the posterior axillary line, and the 7th to 8th intercostal space of the subscapular angle line. Encapsulated effusion can be identified by percussion combined with X-ray or ultrasonography (4)<br>3. marked the puncture point is on the skin (2)	10		
	Disinfection, toweling	Disinfection, toweling, and wearing gloves are in the correct order	5		
	analgesia	1. 2% lidocaine 4-6ml (2)<br>2. The upper border of the rib below the puncture point (4)<br>3. Infiltration anesthesia from the skin to the pleural parietal layer (4)	10		
	puncture	1. Clamp the rubber tube connected after the puncture needle (4)<br>2. The surgeon fixes the skin of the puncture site with his left hand, and slowly pierces the puncture needle in the anesthesia place with	10		

		his right hand.			
	aspiration of effusion	Connect the syringe to the rubber tube, release the clip, and aspirate the pleural effusion. After the pump is full, first clamp the rubber tube, then remove the syringe and discharge the liquid; repeat (the sequence is correct, no pneumothorax)	10		
	Pull the needle	Pull out the puncture needle, press the puncture point with sterile gauze for a while, sterilize the puncture point and cover it with sterile gauze, fix it with tape, and instruct the patient to lie still (in correct order, no pneumothorax)	10		
	Specimen processing	Draw out a pre-measured pleural effusion, measure and send for testing	5		
	Aseptic	no contamination or<br>with contamination , but can be remedied in a timely and correct manner.	5		
Notes and ask questions	Consideration of patients	Explain the purpose of the puncture to the patient before the operation to eliminate concerns; for those who are nervous, drug sedation can be used	5		
	preoperative examination	check the results of coagulation time and platelet count; record blood pressure, pulse, and respiration before operation et al	5		
	pumping limit	No more than 600ml for the first time, and no more than 1000ml each time	5		
	Pleural reaction management	When a pleural reaction occurs, the operation should be stopped immediately, and the patient should be given supine oxygen and fluid supplementation, and epinephrine should be given to maintain blood pressure in the event of shock.	5		
total			100		

assessment teacher: **Inspector:** **time:** **y** **m** **d**

FROM 2 DOPS – Evaluation form

**Assessment objects**	**Assessment results**								
	**need strengthened**			**Reach standard**			**excellent**		
	**1**	**2**	**3**	**4**	**5**	**6**	**7**	**8**	**9**
1.Demonstrates understanding of indications									
2.Commununacate<br>communication skills and Consideration of patient and professionalism									
3.Pre- procedure<br>Obtains informed consent and demonstrates appropriate preparation pre-procedure									
4.Procedure<br>technical ability as applicable to procedure、appropriate analgesia or safe sedation、aseptic technique et.al									
5. Post- procedure									
6.over ability to perform procedure									
total									

assessment teacher： **Inspector：** **time：** **y** **m** **d**

Final score：PART 1 score * 70% + (PART 2 score / 9) * 100 * 30%

FRO example:

FROM 1 胸膜腔穿刺操作评分表 Evaluation form

name: A　　　　　score: 84

object		scoring criteria	Total score	result	Reason for deduction
Pre-	For patient	Measure vital signs, explain relevant content, sign informed consent	5	10	
	For material	Thoracentesis bag, medicines (Aner iodine or iodine-alcohol, 2% lidocaine, (Valium, 0.1% epinephrine)), others such as test tubes, sterile gloves	5		
	For operator	Wash hands, wear hat and mask, understand the patient's condition, the purpose of the puncture, and the chest X-ray et al.	5		
procedure	puncture point	1. Body position: a. Sitting position: facing the back of the chair, with the forearm on the back of the chair, and the forehead lying on the forearm; b. Semi-recumbent position: lying on the back on a high slope, slightly turning the affected side to the healthy side, and raising the pillow on the forearm on the affected side (4)<br>2. Puncture point: usually the 5th intercostal space of the anterior axillary line, the 6th to 7th intercostal space of the midaxillary line, the 7th to 8th intercostal space of the posterior axillary line, and the 7th to 8th intercostal space of the subscapular angle line. Encapsulated effusion can be identified by percussion combined with X-ray or ultrasonography (4)<br>3. marked the puncture point is on the skin (2)	10	8	
	Disinfection, toweling	Disinfection, toweling, and wearing gloves are in the correct order	5	4	
	analgesia	1. 2% lidocaine 4-6ml (2)<br>2. The upper border of the rib below the puncture point (4)<br>3. Infiltration anesthesia from the skin to the pleural parietal layer (4)	10	8	
	puncture	1. Clamp the rubber tube connected after the puncture needle (4)<br>2. The surgeon fixes the skin of the puncture site with his left hand, and slowly pierces the	10	8	

		puncture needle in the anesthesia place with his right hand.			
	aspiration of effusion	Connect the syringe to the rubber tube, release the clip, and aspirate the pleural effusion. After the pump is full, first clamp the rubber tube, then remove the syringe and discharge the liquid; repeat (the sequence is correct, no pneumothorax)	10	8	
	Pull the needle	Pull out the puncture needle, press the puncture point with sterile gauze for a while, sterilize the puncture point and cover it with sterile gauze, fix it with tape, and instruct the patient to lie still (in correct order, no pneumothorax)	10	6	
	Specimen processing	Draw out a pre-measured pleural effusion, measure and send for testing	5	4	
	Aseptic	no contamination or<br>with contamination , but can be remedied in a timely and correct manner.	5	4	
Notes and ask questions	Consideration of patients	Explain the purpose of the puncture to the patient before the operation to eliminate concerns; for those who are nervous, drug sedation can be used	5	3	
	preoperative examination	check the results of coagulation time and platelet count; record blood pressure, pulse, and respiration before operation et al	5	4	
	pumping limit	No more than 600ml for the first time, and no more than 1000ml each time	5	5	
	Pleural reaction management	When a pleural reaction occurs, the operation should be stopped immediately, and the patient should be given supine oxygen and fluid supplementation, and epinephrine should be given to maintain blood pressure in the event of shock.	5	4	
total			100	84	

assessment teacher: **Inspector:** **time:** **y** **m** **d**

FROM 2 DOPS - Evaluation form

name: A score: 7

Assessment objects	Assessment results								
	need strengthened			Reach standard			excellent		
	1	2	3	4	5	6	7	8	9
1.Demonstrates understanding of indications						★			
2.Commununacate communication skills and Consideration of patient and professionalism									★
3.Pre- procedure Obtains informed consent and demonstrates appropriate preparation pre-procedure								★	
4.Procedure technical ability as applicable to procedure、appropriate analgesia or safe sedation、aseptic technique et.al						★			
5. Post- procedure					★				
6.over ability to perform procedure								★	
total	(6+9+8+6+5+8) * 6 = 7								

assessment teacher: **Inspector:** **time:** **y** **m** **d**

Final score: 84 * 70% + (7 / 9) * 100 * 30% = 58.8 + 23.33 = 82.13
